# Manually annotated and curated Dataset of diverse Weed Species in Maize and Sorghum for Computer Vision

**DOI:** 10.1038/s41597-024-02945-6

**Published:** 2024-01-23

**Authors:** Nikita Genze, Wouter K. Vahl, Jennifer Groth, Maximilian Wirth, Michael Grieb, Dominik G. Grimm

**Affiliations:** 1 Technical University of Munich, TUM Campus Straubing for Biotechnology and Sustainability, Bioinformatics, Schulgasse 22, 94315 Straubing, Germany; 2 Weihenstephan-Triesdorf University of Applied Sciences, Bioinformatics, Petersgasse 18, 94315 Straubing, Germany; 3 Institute for Crop Science and Plant Breeding, Bavarian State Research Center for Agriculture, Am Gereuth 6, 85354 Freising, Germany; 4 Technology and Support Centre in the Centre of Excellence for Renewable Resources (TFZ), Schulgasse 18, 94315 Straubing, Germany; 5 Technical University of Munich, TUM School of Computation, Information and Technology (CIT), Boltzmannstr. 3, 85748 Garching, Germany

**Keywords:** Plant breeding, Agroecology, Biodiversity, Machine learning, Image processing

## Abstract

Sustainable weed management strategies are critical to feeding the world’s population while preserving ecosystems and biodiversity. Therefore, site-specific weed control strategies based on automation are needed to reduce the additional time and effort required for weeding. Machine vision-based methods appear to be a promising approach for weed detection, but require high quality data on the species in a specific agricultural area. Here we present a dataset, the Moving Fields Weed Dataset (MFWD), which captures the growth of 28 weed species commonly found in sorghum and maize fields in Germany. A total of 94,321 images were acquired in a fully automated, high-throughput phenotyping facility to track over 5,000 individual plants at high spatial and temporal resolution. A rich set of manually curated ground truth information is also provided, which can be used not only for plant species classification, object detection and instance segmentation tasks, but also for multiple object tracking.

## Background & Summary

Weeds are plants that, although not specifically cultivated, are adapted to grow on arable land. Typically, weeds are considered to be an undesirable element in crop production. Their negative impact on crop development can be described in terms of competition with the crop for resources (nutrients, sunlight, space and water), reduction in productivity, increased challenges during harvesting and an overall increase in the cost of agricultural production. In addition, weeds can be hosts for insects and diseases^1,2^, which might further increase the necessity for control strategies. Nevertheless, weeds might also have positive effects on biodiversity^3^ and soil structure^4^. Therefore, only highly competitive and invasive weed species should be removed which might lead to more sustainable agriculture^5^.

Over centuries, several crop management strategies were established to mitigate the negative impact of weeds, which can be divided into five main categories^6^: ‘preventative’ (preventing weeds from establishing), ‘cultural’ (maintaining field hygiene with low weed seed bank), ‘mechanical’ (removing weeds by mowing, mulching or tilling), ‘biological’ (using natural enemies such as insects or animals), and ‘chemical’ (applying herbicides). Disadvantages of these approaches include financial burden, additional time and effort to varying degrees. In addition, control treatments may impact the health of people, plants, soil, animals, and the environment^7–9^.

**Fig. 1:**
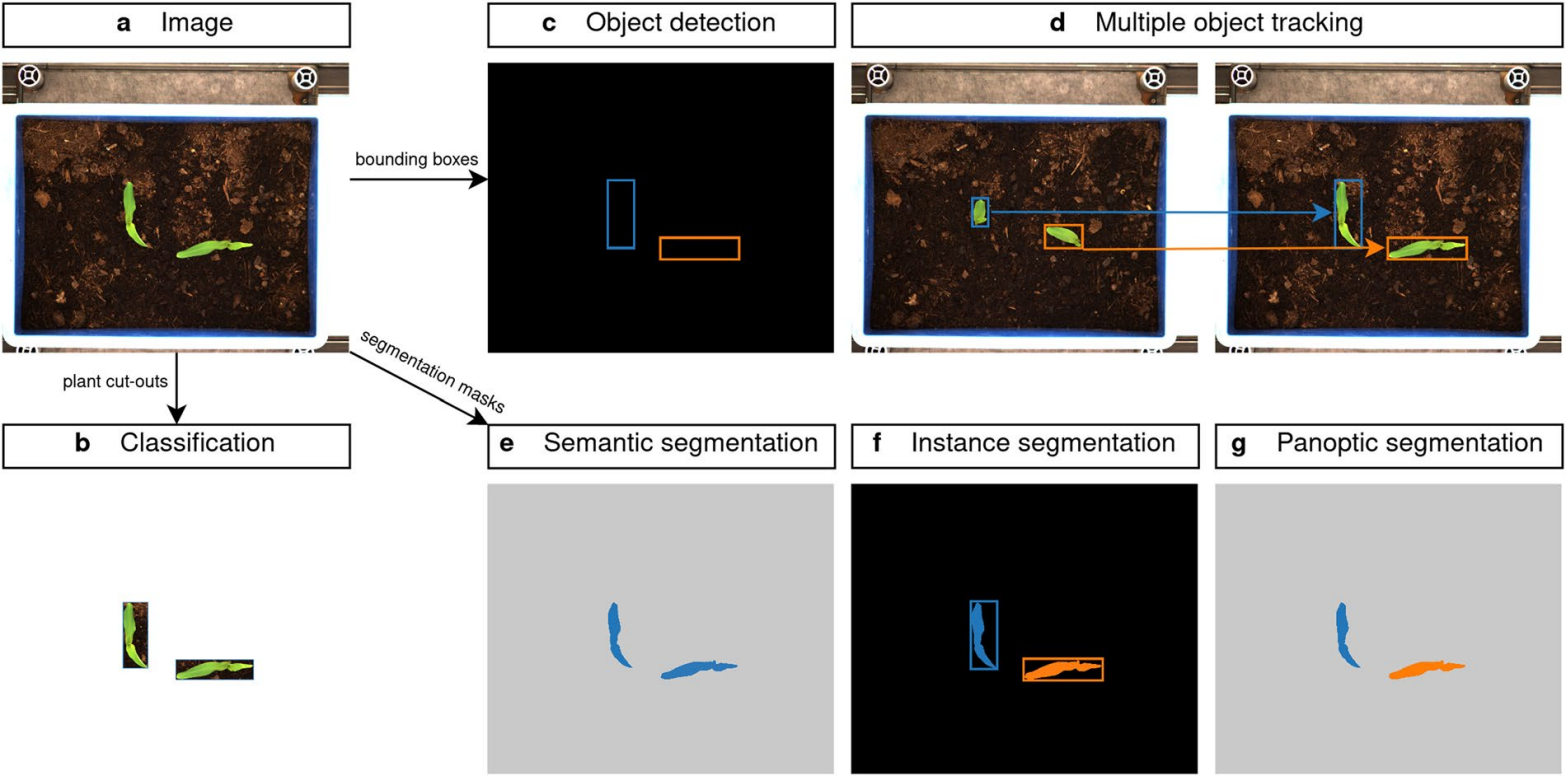
Comparison of different computer vision tasks used in plant phenotyping. Plants (**a**) can be classified (**b**) as individual cut-outs, detected by bounding boxes (**c**), tracked through time (**d**), or segmented containing pixel-wise information. This can be done either semantically (**e**), by instance (**f**) or by the combination of both called panoptic segmentation (**g**).

Sustainable strategies for managing weeds are critical to feed the world’s population while conserving the ecosystems and biodiversity^9^. The limited and rational use of herbicides is an important principle of sustainable farming, as spraying of herbicides leads to waste and can pollute soil and water sources. Furthermore, agrochemical residues are one of the most important food-related concerns. Therefore, additional non-chemical and site-specific weed management (SSWM) strategies^10^ are needed, which should be linked with the farm management system. One key aspect is to automatically and precisely detect weeds to mitigate the additional time and effort for either site-specific or weed-specific herbicide application or mechanical weed control. Numerous studies demonstrated methods to automatically detect weeds on the field or in greenhouses, where computer vision-based methods seem the most promising^11–19^. These methods can be grouped into different tasks with varying ground truth information, as shown in Fig. 1. Starting from an image as shown in Fig. 1a, image classification is the least accurate task (Fig. 1b). It is applied to a single plant cut-out without location and thus cannot be applied in SSWM tasks, where the simultaneous detection and localization of multiple plants is desired. Therefore, the object detection^20^ task might be utilized. However, this task detects rectangular bounding boxes (Fig. 1c) which is not satisfactory due to the complex and irregular shape of the plants. Also, these models are prone to occlusion^21^ which diminishes their performance in areas with high weed infestation^22^. Nevertheless, the analysis of a plant’s growth dynamics can be achieved, where multiple objects are tracked through time (see Fig. 1d). Moreover, by using segmentation masks, additional tasks can be performed. Here, by convention any countable entity (i.e. plant, person, etc.) is named ‘*thing*’ and any uncountable region (i.e. soil, sky) is called ‘*stuff*’: The semantic segmentation task^23^ provides a precise delineation of *stuff*, which separates every pixel in the image by class label, but cannot separate different plants of the same class^24^ (see Fig. 1e). This is crucial in selective weed management^25^ to conserve biodiversity by removing only competitive weeds. Consequently, instance segmentation^24^ (Fig. 1f) can generate accurate detections of *things* individually, which can be used in many downstream tasks such as weed density assessment or biomass estimation^26^. Nevertheless, this type of model is only able to detect countable objects (*things*) and does not consider *stuff* regions. Finally, panoptic segmentation^27^ combines the concept of both semantic and instance segmentation and assigns two labels (semantic label and instance id) to each pixel in an image (Fig. 1g).

The basis for the development, validation and assessment of such systems is the availability of high quality data on weed diversity in a particular area of interest. Several datasets are publicly available, but lack several aspects for precise plant phenotyping, as summarized in Table 1.

**Table 1:** Comparison of ground-based image datasets for plant detection.

Dataset name	Labeled samples	Classification	Object detection	Semantic segmentation	Instance segmentation	Multiple object tracking (growth)
Open Plant Phenotype Database (OPPD)^28^	315,038	✓	✓			✓
Pl@ntNet-300k^29^	306,146	✓				
**MFWD (ours)**^30^	**200,148**	✓	✓	✓	✓	✓
Plant Village Dataset^41^	54,303	✓				
Deep Seedling^42^	33,444	✓	✓			
DeepWeeds^43^	17,509	✓				
Weed25^44^	14,023	✓	✓			
WE3DS^45^	11,544	✓	✓	✓		
Sudars *et al*.^46^	7,853	✓	✓			
Plant Seedling^47^	5,539	✓	✓			✓
Champ *et al*.^24^	2,489	✓	✓	✓	✓	
Ladybird Cobbitty 2017 Brassica Dataset^48^	2,245	✓	✓	✓		
CWFID^49^	494	✓	✓			
Leaf Segmentation and Counting Challenge^50^	284		✓	✓	✓	✓

Most datasets available lack variability in the data (low number of individuals or plant species) limiting their usability in different studies. Only a few datasets are larger than 100,000 annotated plant samples, including Open Plant Phenotype Database (OPPD)^28^ and Pl@ntNet-300k^29^. However, Pl@ntNet-300k can only be used for classification tasks without tracking plant growth stages and OPPD is missing semantic and instance segmentation masks, which are important for precise phenotyping. Also, the bounding box information of a plant is often not sufficient, as it is too coarse for most weed management applications. Therefore, semantic segmentation or even more accurate instance segmentation masks are required. Finally, tracking a plant over time provides valuable insight into growth dynamics.

In this work we have created a high-quality dataset of different plant species with a high temporal and spatial resolution. We added manually curated semantic and instance segmentation masks of a subset to make this dataset suitable for weed management tasks. For this purpose, we used a high throughput phenotyping system to ensure a high degree of automation, as this system was equipped with controlled illumination and an automatic irrigation system. In our dataset, we included images of plants captured multiple times per day. This included captures in the evening, when the appearance of some species changes due to their dependency on sunlight. In addition, we generated data from different varieties of sorghum and maize, focusing on a wide range of seedling weeds that are also common in agricultural sites where these crops are grown.

## Methods

**Fig. 2:**
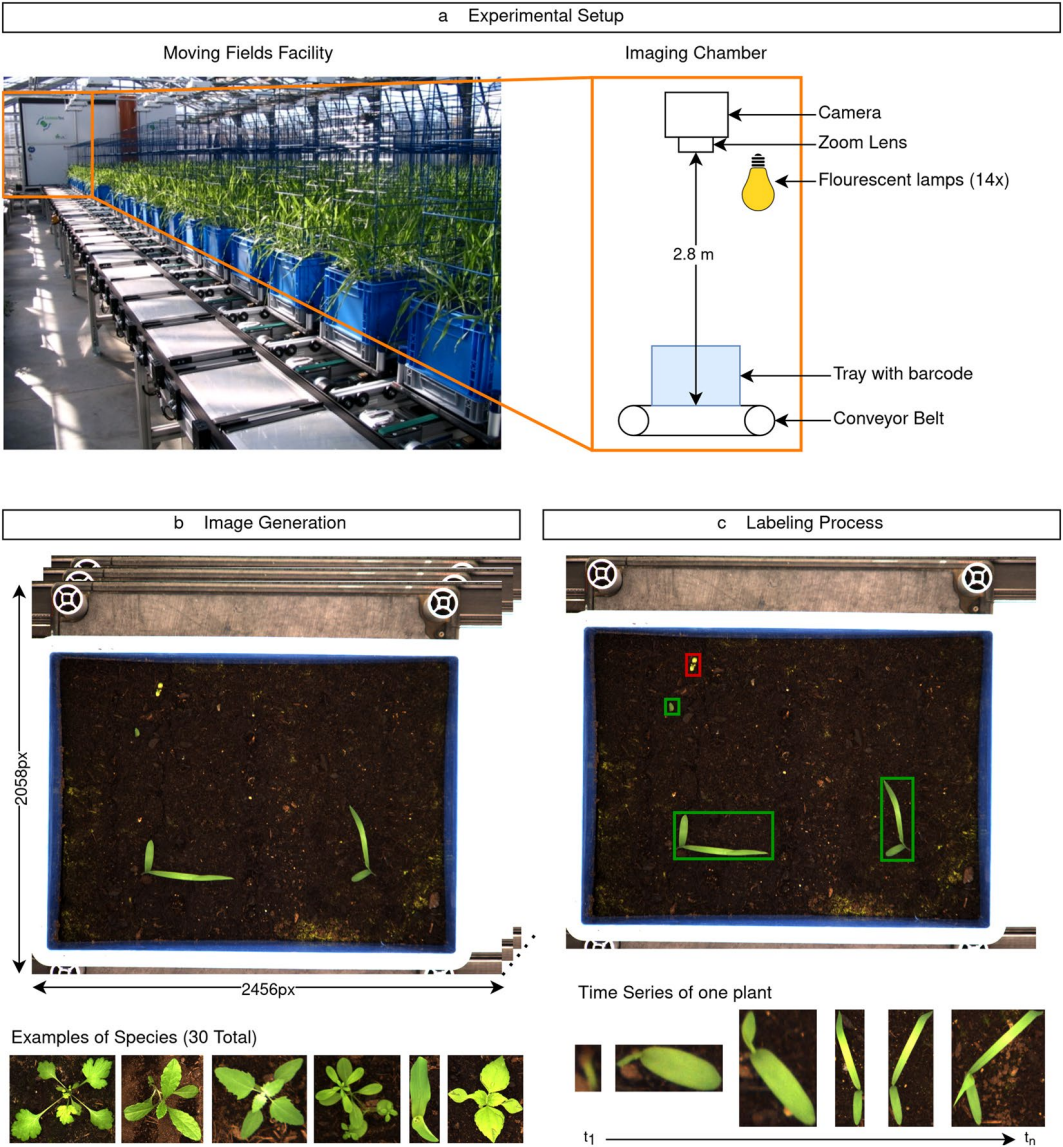
Outline of the experimental workflow. (**a**) Experimental setup using an automated phenotyping facility. (**b**) Generation of images with high temporal and spatial resolution of 30 different plant species. (**c**) Labeling process illustrated by bounding box information for the object detection task (weed illustrated in red and sorghum plants in green).

The methodology can be summarized into three steps, as shown in Fig. 2. First, we will describe the experimental setup (Fig. 2a). Second, we will illustrate the image generation (Fig. 2b) and conclude with the labeling process (Fig. 2c).

### Experimental Setup

To generate a dataset consisting of high-quality images that capture the initial growth dynamics of individual plants of several weed species, a greenhouse experiment was performed at the Moving Fields facility (https://www.lfl.bayern.de/verschiedenes/ueberuns/272457/index.php) of the Bavarian State Research Center for Agriculture in Freising, Germany. In the experiment, plants were grown in micro-plots that were watered and photographed automatically on at least a daily basis from the day of sowing until harvest, which took place at around shooting. Built into a greenhouse, the Moving Fields facility (LemnaTec GmbH) consists of a conveyor belt system, three irrigation stations and four ‘Scanalyzer 3d’ photo cabins, which together enable experimental units consisting of plants growing on micro-plots to be automatically moved, watered, weighted and photographed. The greenhouse can be climatically controlled with regard to humidity and temperature and can be illuminated by 48 sodium-vapor lamps (Philips Son-T AGRO). The conveyor belt system (Bosch Rexroth TS2plus) accommodates and enables the movement of 390 carriers (micro-plots). At the three measuring stations, digital scales (Bizerba ST) and high-pressure pumps (Wartson-Marlow) enable carriers, together with any plants transported by them, to be weighed and to be watered to a unit-specific target weight. The plant species included in the dataset, listed in Table 2 and Table 3, were selected as weed species common to fields of sorghum grown in Germany. Additional selection criteria were 1) commercial availability and 2) the ability to be grown at the climatically controlled conditions of a greenhouse. Seeds of the species involved were acquired from commercial breeders in Germany, the Netherlands and France. All plants were grown in boxes of size 40 × 30 × 22 cm (outer dimensions). The color of these boxes was blue, to facilitate image analysis afterwards. Each box was filled to about half height (roughly 11 cm after compression) with a commercial peat-free substrate (Höfter GmbH), primarily consisting of coconut fibers. Plants were grown as monocultures; each box contained plants of one species only. To yield data for enough individual plants per species, the number of boxes varied between species due to different germination rates.

**Table 2:** Selected weed species with corresponding amount of data captured and labeled.

EPPO Code	Dicot	Botanical Name	# Images	Captured Trays	Labeled Trays for		
					classification	detection	segmentation
ACHMI	✓	*Achillea millefolium*	2,893	25	25	5	0
AGRRE		*Elymus repens*	2,848	29	12	12	0
ALOMY		*Alopecurus myosuroides*	3,595	28	11	11	0
ARTVU	✓	*Artemisia vulgaris*	2,881	34	34	18	1
CHEAL	✓	*Chenopodium album*	1,652	23	23	9	1
CIRAR	✓	*Cirsium arvense*	2,546	15	15	15	1
CONAR	✓	*Convolvulus arvensis*	4,914	27	27	27	1
ECHCG		*Echinochloa crus-galli*	2,622	27	25	25	0
GALAP	✓	*Galium aparine*	1,513	23	23	14	1
GASPA	✓	*Galinsoga parviflora*	847	13	13	13	1
LAMAL	✓	*Lamium album*	5,276	36	36	32	0
MATCH	✓	*Matricaria chamomilla*	1,681	21	21	8	0
PLAMA	✓	*Plantago major*	7,856	43	43	37	0
POAAN		*Poa annua*	2,574	23	11	11	0
POLCO	✓	*Fallopia convolvulus*	330	9	9	9	1
POROL	✓	*Portulaca oleracea*	4,435	21	21	5	1
PULDY	✓	*Pulicaria dysenterica*	1,745	21	21	10	1
SOLNI	✓	*Solanum nigrum*	1,472	22	22	12	1
SSYOF	✓	*Sisymbrium officinale*	2,477	14	14	10	0
STEME	✓	*Stellaria media*	1,887	22	22	8	0
THLAR	✓	*Thlaspi arvense*	4,044	38	38	36	1
VEROF	✓	*Veronica officinalis*	3,987	22	22	5	0
VIOAR	✓	*Viola arvensis*	1,261	17	17	11	1

**Table 3:** Selected crop varieties with corresponding amount of data captured and labeled.

Abbreviation	EPPO Code	Variety	# Images	Captured Trays	Labeled Trays for		
					classification	detection	segmentation
SORFR	SORVU	KWS Freya	2,881	27	27	27	0
SORHA	SORVU	KWS Hannibal	552	27	5	5	1
SORKM	SORVU	KWS Merlin	511	27	5	5	0
SORSA	SORVU	KWS Sammos	1,279	27	12	12	0
SORKS	SORVU	KWS Sole	604	27	5	5	0
SORRS	SORVU	RAGT Swingg	447	27	5	5	0
ZEALP	ZEAMX	Lidea Palladium	930	38	38	38	0
ZEAKJ	ZEAMX	KWS Johaninio	1,660	38	38	38	1

The number of seeds planted in each box was made dependent on the expected germination rate, which was adjusted throughout the experiment. Thus, seed density varied both within species over time and between species. Following breeder recommendations, some seeds were kept in a vernalization room (at 4 °C) or treated with gibberellin acid (GA_3_) to ensure germination success. Units were sent to an automatic watering station as often as frequent imaging allowed, in practice at least twice a day. Each unit was watered to its unique target weight. This target weight initially corresponded to the unit’s weight at sowing. Throughout the experiment, target weights were adjusted repeatedly to prevent boxes from becoming either too dry or too wet. Twice a week, all boxes were examined to score seedling emergence, to thin the standing stock in order to minimize overlap between individual plants and to harvest plants that either started shooting or that became too big for the box they were growing in.

In addition, different varieties of maize (*Zea Mays*) and sorghum (*Sorghum bicolor*) were grown and captured, as shown in Table 3.

### Image Generation

To generate well illuminated, high-resolution top-down images of the experimental units, one of the Scanalyzer 3D imaging cabins of the Moving Fields facility was used. In this cabin, one RGB camera (Basler piA2400-17gm) is mounted 2.8 m perpendicular above the conveyor band. This camera takes images with 2456 × 2058 pixels. This camera is equipped with a motorized zoom lens (Pentax C6Z1218M3-5); throughout the experiment, however, this lens was set fixed at a single position, which resulted in a ground resolution of ∼ 0.17 mm per pixel. The micro-plots were illuminated by 14 fluorescent lamps (Osram HE 28 W/865) that were also mounted perpendicular above the conveyor band. Units were imaged as often as possible, in practice at least once a day on the two days per week on which plant maintenance took place and at least twice a day on all other days. The units were tracked over a development period from sowing till the last plant either started shooting or became too big for the setup, representing the relevant stages for weed control on the field for sorghum. The images collected were stored in a LemnaTec-specific raw format, after which they were converted to PNG format. Each experimental unit was marked with a unique numerical barcode composed of identity codes for 1) the species, 2) the treatment (with or without GA3) and 3) the replicate at issue. Each image was saved with the barcode of the unit that was on the image as well as the date and time of image acquisition.

### Labeling Process

The complete dataset was labeled using the open-source software CVAT (Computer Vision Annotation Tool; https://www.cvat.ai/) as a self-hosted solution. This software made the labeling time-efficient, as it provided an easy interface for multiple object tracking by adding time series information per plant. Each species was labeled individually, providing the EPPO code as a label. Although only one species was seeded per tray, more weed species germinated during the experiment (compare Table 4), as the seed assortment was not completely pure. Therefore, the additional label “weed” was used to annotate these plants and the unknown species was identified by an expert in a second step. The correct species could not be specified for all plants due to several reasons (i.e., little germination resulting in small plants, occlusion with other plants, etc.), especially when they were not part of our initial assortment.

**Table 4:** Additional germinating weed species (dicots) that were not sown explicitly.

EPPO code	Botanical name	# Plant individuals	# Plant samples
AETCY	*Aethusa cynapium*	4	110
GERMO	*Geranium molle*	4	139
POLAM	*Persicaria amphibia*	55	2237
POLAV	*Polygonum aviculare*	2	62
VICVI	*Vicia villosa*	1	32

The instance segmentation masks were manually drawn using another open-source software, GIMP (GNU Image Manipulation Program), which provides pixel-level information. Therefore, we selected one tray from each of 14 plant species, as not all species could be labeled due to complexity and time constraints (see Fig. 1g).

## Data Records

**Fig. 3:**
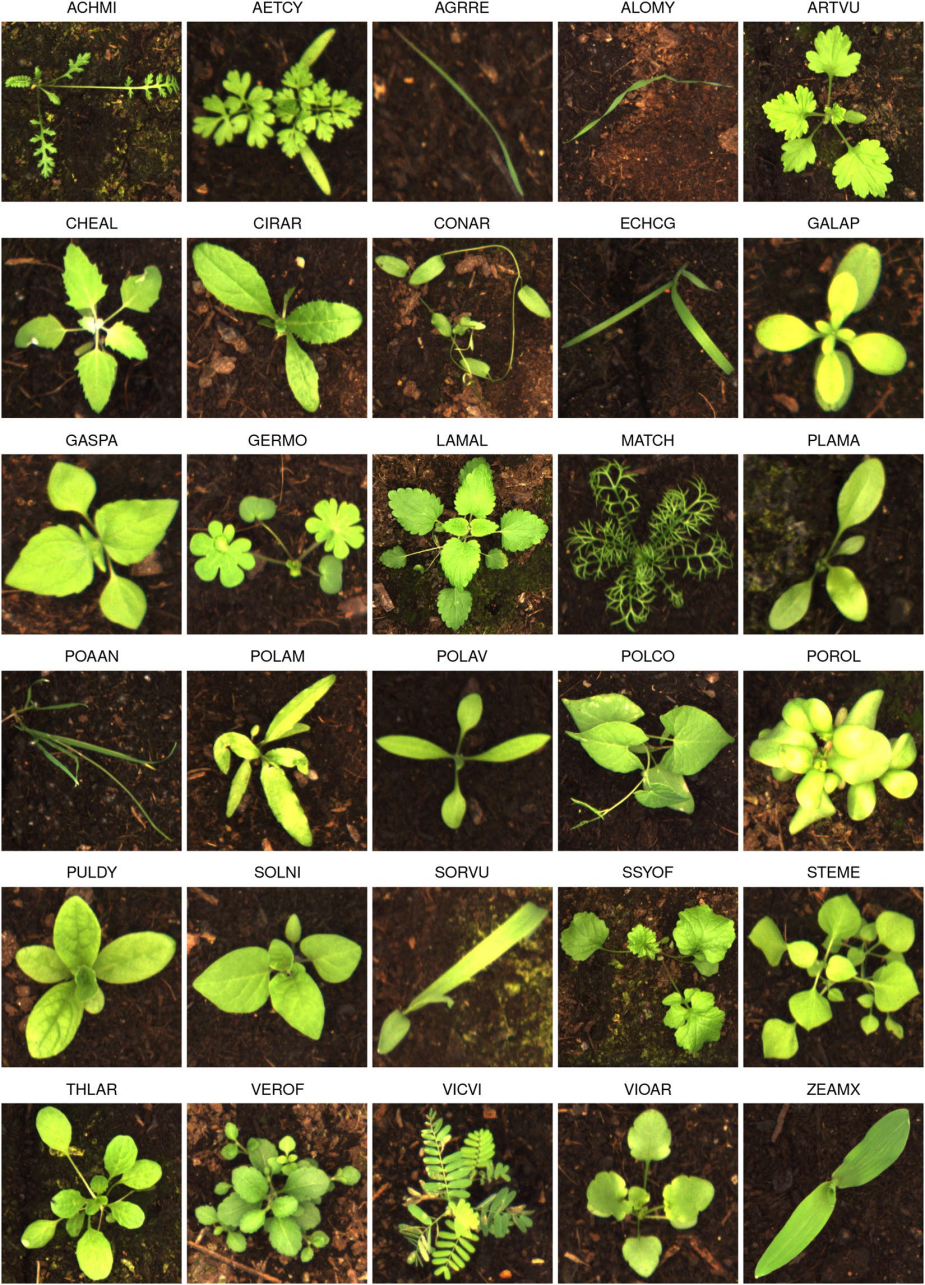
Example of each plant species with corresponding EPPO code.

The Moving Fields Weed Dataset^30^ (MFWD) is deposited at the digital library of the Technical University of Munich (https://mediatum.ub.tum.de/1717366). The dataset consists of 94,321 high temporal and spatial resolution images of 30 different plant species (see Fig. 3).

Additional ground truth data is provided, consisting of the plant species, a bounding box per plant, and time series information to track the same plant individual through growth. We labeled a large subset of these images, resulting in 200,148 records for 5,068 plants in the current version. Additional information is shown in Table 5.

**Table 5:** Summary of the current version of our dataset.

Data Content	Data Statistics
High Resolution Tray Images	94,321
Individual Plant Samples (Object Detection)	200,148
Individual Plant Samples (Instance Segmentation)	2,025
Time Series of individual Plants	5,068
Weed Species	28
Sorghum Varieties	6
Maize Varieties	2
All PNG images [GB]	814
All JPEG images [GB]	114

Image data is stored in PNG format to ensure the highest possible quality without compression artifacts. Compressed (JPEG) images are also stored to ensure accessibility with lower Internet bandwidth. All object detection and object tracking information are stored in a separate CSV file named “gt.csv”. The segmentation masks are stored in the folder “masks”. An additional folder is provided containing all images without ground truth annotations. The contents of the CSV file are explained in Table 6:

**Table 6:** Description of the csv file containing ground truth information.

Column	Explanation
track_id	ID of the individual time series connecting bbox_id of different images in one tray
label_id	ID of the plant species, saved as EPPO code
bbox_id	ID of the individual sample
xmin	left coordinate of the individual sample
ymin	top coordinate of the individual sample
xmax	right coordinate of the individual sample
ymax	bottom coordinate of the individual sample
filename	relative file path to the image
tray_id	ID of the tray

## Technical Validation

The growth experiments were conducted using multiple trays, resulting in different numbers of replicates per species. Here, a minimum of nine replicates were used. Seeds were treated according to breeders’ recommendations. Some weeds germinated only when treated with gibberellin acid. Therefore, the optimal procedure was evaluated in a prior experiment.

The quality of the bounding boxes and labels was ensured twofold. First, a valid bounding box could be evaluated during the labeling process by using tools in CVAT directly and by an additional human inspector doing quality control. Second, using the time-series information, all plant cut-outs of one plant individual could be plotted in a series of images to visually inspect the bounding boxes. Finally, plants of additional not sown species could be assessed and classified in an additional step. Remaining instances were labeled as class “Weed”, as they were tiny and thus could not be labeled by species.

**Fig. 4:**
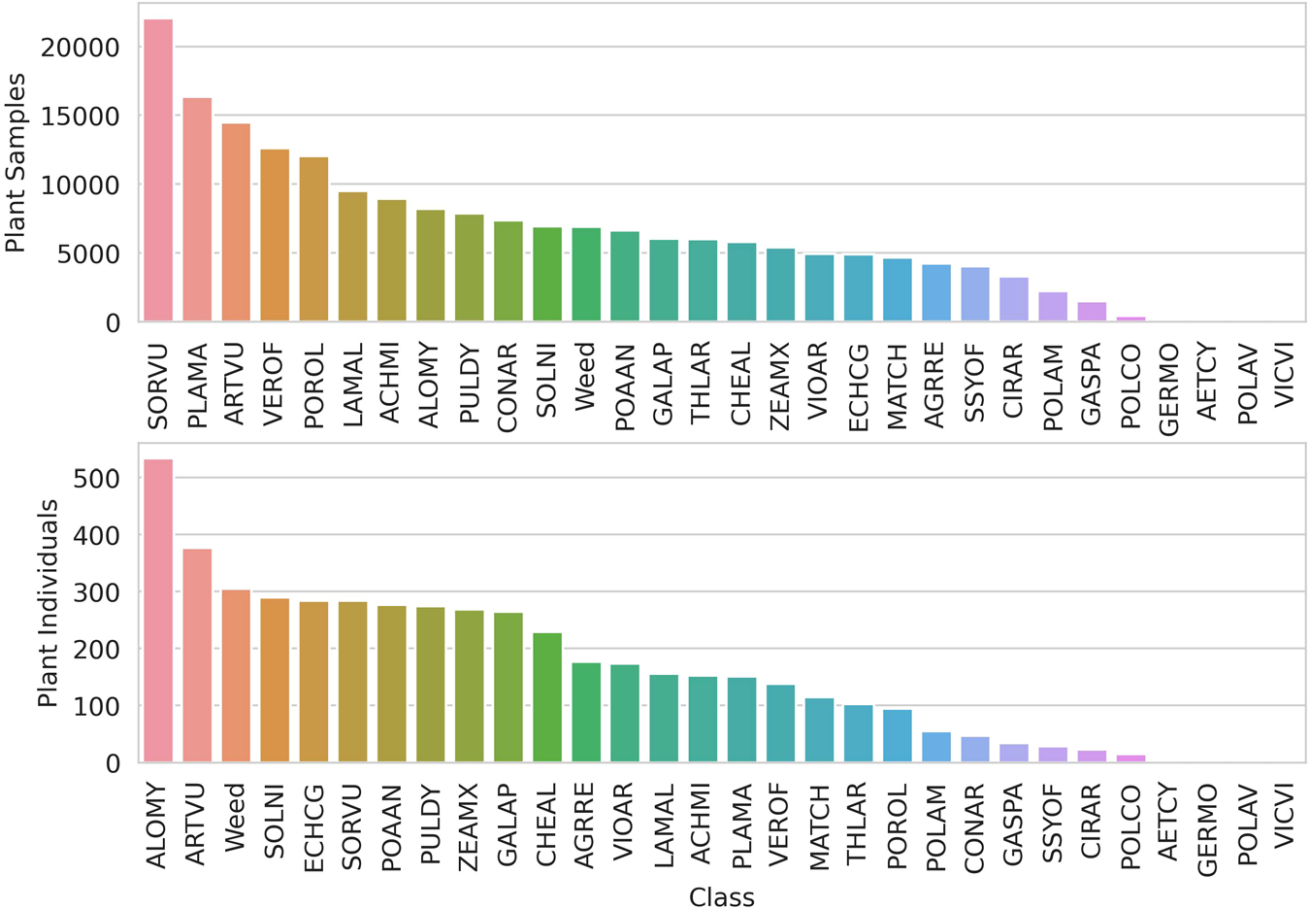
Distribution of plant samples (separate images) and plant individuals (multiple images of one plant through time) per class.

The high variability in the seed germination rate of different weed species resulted in a very diverse data set of different weeds with different germination rates, as shown in Fig. 4.

## Usage Notes

The dataset can be downloaded via custom Python scripts (https://github.com/grimmlab/MFWD) and used as a resource for precision agriculture, smart farming, and computer vision related tasks. In addition, we encourage the development of algorithms that take plant phenotyping data into account. Since the dataset consists of high-resolution images, we also provide a custom Python script to easily resize the images. The dataset could be a useful resource for the computer science community in general to develop novel machine learning and computer vision algorithms for automatic weed detection. Here, the data could be used in classification as well as object detection and segmentation tasks. Furthermore, the additional time series information makes our dataset suitable for multiple object tracking. Finally, the inherent inter- and intra-class variance can be used to evaluate new algorithms that address class imbalance, which is a challenge in many machine learning tasks^31–33^.

To demonstrate the usefulness of the dataset, we provide a simple baseline experiment on the image classification task. For this purpose, we use plant cut-outs from the jpeg-compressed images and rescale them to an image size of 224 × 224 pixels². Here we focused on the multi-species classification for sorghum, i.e. all sorghum varieties were labeled as “SORVU” and excluded all maize images. Additionally, the generic weed class was excluded for this experiment, as these were mostly small plants which could not even be classified by the human eye. We also excluded POLAV and VICVI from the experiment because they contained less than three plant individuals and thus could not be separated into training, validation, and test sets. We strongly recommend stratifying the data by plant individuals, as the temporal resolution is high, and models may overfit if the data were randomly split. The final dataset of 27 plant species contained 167,505 images and was split into a training- (~60%), validation- (~20%), and test-set (~20%).

We selected two different deep learning-based model architectures, ResNet-10^34^ and EfficientNet_b0^35^, to evaluate the classification performance. For the hyperparameter optimization we used grid-search and five different learning rates (sampled from a log uniform distribution in the range between 1e-3 and 1e-4). We sampled 512 plant cut-outs in a batch by oversampling the minority classes, due to high class imbalances. The networks were initialized with weights from ImageNet. The Adam^36^ optimizer with a learning rate scheduler and the cross-entropy loss^37^ was used to train the models. We validated the models using the validation-set by calculating the weighted f1-score^38,39^, due to the high class imbalance. Each model was trained for a maximum of 50 epochs. Early stopping^40^ was used as a regularization technique to avoid overfitting. After training the models, EfficientNet_b0 with a learning rate of ~5.4*10^−4^ gave the best results on the validation set with an f1-score of 90.00%. The summary of the hyperparameter optimization is shown in Table 7.

**Table 7:** Summary of the hyperparameter optimization calculated on the validation set.

model	learning rate (*10^−4^)	best epoch	f1-score (%)
EfficientNet_b0	2.37	8	88.75
EfficientNet_b0	8.93	1	83.93
**EfficientNet_b0**	**5.40**	**32**	**90.00**
EfficientNet_b0	3.97	8	89.35
EfficientNet_b0	1.43	26	88.88
ResNet-10	2.37	20	88.24
ResNet-10	8.93	1	81.28
ResNet-10	5.40	21	88.74
ResNet-10	3.97	20	88.70
ResNet-10	1.43	20	87.75

Finally, the best performing model was applied to the hold-out test set to evaluate the generalization abilities on an unseen dataset. Here, the model achieved a weighted f1-score of 90.57%, indicating good generalization performance within the MFWD dataset. The complete code for training and testing the model is publicly available in our GitHub repository.

However, deep learning models trained on this dataset may not be applicable to out-of-context data, such as weed detection in drone imagery. Here, pre-training a model on our dataset and fine-tuning it to the target task might be a feasible strategy to scale up weed detection in agricultural landscapes. However, the main target application of our dataset is to encourage the research community to develop new computer vision algorithms on a unified dataset, thus increasing the reproducibility of the results.

## Data Availability

The code to download the dataset is publicly available for download on GitHub: https://github.com/grimmlab/MFWD.

## References

[1] Dentika P, Ozier-Lafontaine H, Penet L (2021). Weeds as Pathogen Hosts and Disease Risk for Crops in the Wake of a Reduced Use of Herbicides: Evidence from Yam (Dioscorea alata) Fields and Colletotrichum Pathogens in the Tropics. J Fungi.

[2] Norris RF, Kogan M (2000). Interactions between weeds, arthropod pests, and their natural enemies in managed ecosystems. Weed Sci.

[3] Schumacher M, Dieterich M, Gerhards R (2020). Effects of weed biodiversity on the ecosystem service of weed seed predation along a farming intensity gradient. Glob Ecol Conserv;.

[4] Logsdon, S. D. Root effects on soil properties and processes: Synthesis and future research needs. In: *Enhancing Understanding and Quantification of Soil-Root Growth Interactions*. John Wiley & Sons, Ltd, pp 173–196, 10.2134/advagricsystmodel4.c8 (2015).

[5] Harker, K. N., Clayton, G. W. & O’Donovan, J. T. Reducing agroecosystem vulnerability to weed invasion. In: *Invasive Plants: Ecological and Agricultural Aspects*. Birkhäuser Basel, pp 195–207, 10.1007/3-7643-7380-6_12 (2005).

[6] Harker KN, O’Donovan JT (2013). Recent Weed Control, Weed Management, and Integrated Weed Management. Weed Technol;.

[7] Myers JP (2016). Concerns over use of glyphosate-based herbicides and risks associated with exposures: A consensus statement. Environ. Heal. A Glob. Access Sci. Source..

[8] Steinmetz Z (2016). Plastic mulching in agriculture. Trading short-term agronomic benefits for long-term soil degradation?. Sci. Total Environ.;.

[9] MacLaren C, Storkey J, Menegat A, Metcalfe H, Dehnen-Schmutz K (2020). An ecological future for weed science to sustain crop production and the environment. A review. Agron. Sustain. Dev..

[10] Christensen S (2009). Site-specific weed control technologies. Weed Res;.

[11] Hasan, A. S. M. M., Sohel, F., Diepeveen, D., Laga, H. & Jones, M. G. K. A survey of deep learning techniques for weed detection from images. Comput. Electron. Agric.; **184**, 10.1016/j.compag.2021.106067 (2021).

[12] Wang A, Zhang W, Wei X (2019). A review on weed detection using ground-based machine vision and image processing techniques. Comput. Electron. Agric.;.

[13] Dian Bah, M., Hafiane, A. & Canals, R. Deep learning with unsupervised data labeling for weed detection in line crops in UAV images. *Remote Sens;* **10**, 10.3390/rs10111690 (2018).

[14] Bakhshipour A, Jafari A (2018). Evaluation of support vector machine and artificial neural networks in weed detection using shape features. Comput. Electron. Agric.;.

[15] Sivakumar, A. N. V. *et al*. Comparison of object detection and patch-based classification deep learning models on mid-to late-season weed detection in UAV imagery. *Remote Sens;* **12**, 10.3390/rs12132136 (2020).

[16] Genze, N. *et al*. Improved weed segmentation in UAV imagery of sorghum fields with a combined deblurring segmentation model. *Plant Methods*; **19**. 10.1186/s13007-023-01060-8 (2023). 10.1186/s13007-023-01060-8 PMC10463442 37608384

[17] Genze, N. *et al*. Deep learning-based early weed segmentation using motion blurred UAV images of sorghum fields. *Comput. Electron. Agric.;* **202**, 10.1016/j.compag.2022.107388 (2022).

[18] Milioto, A., Lottes, P. & Stachniss, C. Real-Time Semantic Segmentation of Crop and Weed for Precision Agriculture Robots Leveraging Background Knowledge in CNNs. In: *Proceedings - IEEE International Conference on Robotics and Automation*. Institute of Electrical and Electronics Engineers Inc., pp 2229–2235, 10.1109/ICRA.2018.8460962 (2018).

[19] Lottes, P., Khanna, R., Pfeifer, J., Siegwart, R. & Stachniss, C. UAV-based crop and weed classification for smart farming. In: *Proceedings - IEEE International Conference on Robotics and Automation*. Institute of Electrical and Electronics Engineers Inc., pp 3024–3031, 10.1109/ICRA.2017.7989347 (2017).

[20] Zhao ZQ, Zheng P, Xu ST, Wu X (2019). Object Detection with Deep Learning: A Review. IEEE Trans. Neural Networks Learn. Syst.;.

[21] Genze, N., Bharti, R., Grieb, M., Schultheiss, S. J. & Grimm, D. G. Accurate machine learning-based germination detection, prediction and quality assessment of three grain crops. *Plant Methods*; **16**. 10.1186/s13007-020-00699-x (2020). 10.1186/s13007-020-00699-x PMC7754596 33353559

[22] Janneh, L. L., Zhang, Y., Cui, Z. & Yang, Y. Multi-level feature re-weighted fusion for the semantic segmentation of crops and weeds. *J King Saud Univ - Comput Inf Sci*; **35**, 10.1016/j.jksuci.2023.03.023 (2023).

[23] Long, J., Shelhamer, E. & Darrell, T. Fully convolutional networks for semantic segmentation. In: *Proceedings of the IEEE Computer Society Conference on Computer Vision and Pattern Recognition*. IEEE Computer Society, pp 431–440, 10.1109/CVPR.2015.7298965 (2015).

[24] Champ, J. *et al*. Instance segmentation for the fine detection of crop and weed plants by precision agricultural robots. *Appl Plant Sci*; **8**. 10.1002/aps3.11373 (2020). 10.1002/aps3.11373 PMC7394709 32765972

[25] von Redwitz, C. *et al*. Better-Weeds – Next generation weed management. *Tagungsband 30 Dtsch Arbeitsbesprechung über Frag der Unkrautbiologie und -bekämpfung*; 432–437, 10.5073/20220124-075254 (2022).

[26] Sapkota, B. B. *et al*. Use of synthetic images for training a deep learning model for weed detection and biomass estimation in cotton. *Sci Rep*; **12**. 10.1038/s41598-022-23399-z (2022). 10.1038/s41598-022-23399-z PMC9666527 36379963

[27] Kirillov, A., He, K., Girshick, R., Rother, C. & Dollar, P. Panoptic segmentation. In: *Proceedings of the IEEE Computer Society Conference on Computer Vision and Pattern Recognition*. IEEE Computer Society, pp 9396–9405, 10.1109/CVPR.2019.00963 (2019).

[28] Madsen, S. L. *et al*. Open plant phenotype database of common weeds in Denmark. *Remote Sens;* **12**. 10.3390/RS12081246 (2020).

[29] Garcin, C., Joly, A., Bonnet, P., Chouet, M. & Servajean, M. Pl@ntNet-300K: a plant image dataset with high label ambiguity and a long-tailed distribution. *NeurIPS 2021-35th Conf Neural Inf Process Syst* 2021.

[30] Genze N (2023). Technical University of Munich, mediaTUM.

[31] Chou, H. P., Chang, S. C., Pan, J. Y., Wei, W. & Juan, D. C. Remix: Rebalanced Mixup. In: *Lecture Notes in Computer Science*. Springer Science and Business Media Deutschland GmbH, pp 95–110, 10.1007/978-3-030-65414-6_9 (2020).

[32] Zhou, F., Yang, S., Fujita, H., Chen, D. & Wen, C. Deep learning fault diagnosis method based on global optimization GAN for unbalanced data. *Knowledge-Based Syst*; **187**. 10.1016/j.knosys.2019.07.008 (2020).

[33] Kaur, H., Pannu, H. S. & Malhi, A. K. A systematic review on imbalanced data challenges in machine learning: Applications and solutions. ACM Comput. Surv.; **52**. 10.1145/3343440 (2019).

[34] He, K., Zhang, X., Ren, S. & Sun, J. Deep residual learning for image recognition. In: *Proceedings of the IEEE Computer Society Conference on Computer Vision and Pattern Recognition*, pp 770–778, 10.1109/CVPR.2016.90 (2016).

[35] Tan, M. & Le, Q. V. EfficientNet: Rethinking model scaling for convolutional neural networks. In: *36th International Conference on Machine Learning, ICML* 2019. 2019, pp 10691–10700.

[36] Kingma, D. & Ba, J. Adam: A Method for Stochastic Optimization. In: *International Conference on Learning Representations (ICLR)*. San Diega, CA, USA, 2015.

[37] Cox DR (1958). The Regression Analysis of Binary Sequences. J R Stat Soc Ser B.

[38] Chinchor, N. MUC-4 evaluation metrics. *4th Messag Underst Conf MUC 1992 - Proc*: 22–29, 10.3115/1072064.1072067 (1992).

[39] He H, Garcia EA (2009). Learning from Imbalanced Data. IEEE Trans Knowl Data Eng.

[40] Yao Y, Rosasco L, Caponnetto A (2007). On early stopping in gradient descent learning. Constr Approx.

[41] Hughes, D. P. & Salathe, M. An open access repository of images on plant health to enable the development of mobile disease diagnostics. *arXiv.org* 2015.http://arxiv.org/abs/1511.08060 (accessed 27 Oct2023).

[42] Jiang, Y., Li, C., Paterson, A. H. & Robertson, J. S. DeepSeedling: Deep convolutional network and Kalman filter for plant seedling detection and counting in the field. *Plant Methods*; **15**. 10.1186/s13007-019-0528-3 (2019). 10.1186/s13007-019-0528-3 PMC6874826 31768186

[43] Olsen, A. *et al*. DeepWeeds: A Multiclass Weed Species Image Dataset for Deep Learning. *Sci Rep*; **9**. 10.1038/s41598-018-38343-3 (2019). 10.1038/s41598-018-38343-3 PMC6375952 30765729

[44] Wang, P. *et al*. Weed25: A deep learning dataset for weed identification. *Front Plant Sci*; **13**. 10.3389/fpls.2022.1053329 (2022). 10.3389/fpls.2022.1053329 PMC9748680 36531369

[45] Kitzler, F., Barta, N., Neugschwandtner, R. W., Gronauer, A. & Motsch, V. WE3DS: An RGB-D Image Dataset for Semantic Segmentation in Agriculture. *Sensors*; **23**. 10.3390/s23052713 (2023). 10.3390/s23052713 PMC10007111 36904917

[46] Sudars, K., Jasko, J., Namatevs, I., Ozola, L. & Badaukis, N. Dataset of annotated food crops and weed images for robotic computer vision control. *Data Br*; **31**. 10.1016/j.dib.2020.105833 (2020). 10.1016/j.dib.2020.105833 PMC7305380 32577458

[47] Giselsson, T. M., Jørgensen, R. N., Jensen, P. K., Dyrmann, M. & Midtiby, H. S. A Public Image Database for Benchmark of Plant Seedling Classification Algorithms. *arXiv.org* 2017.http://arxiv.org/abs/1711.05458 (accessed 27 Oct2023).

[48] Bender A, Whelan B, Sukkarieh S (2020). A high-resolution, multimodal data set for agricultural robotics: A Ladybird’s-eye view of Brassica. J F Robot;.

[49] Haug, S. & Ostermann, J. A crop/weed field image dataset for the evaluation of computer vision based precision agriculture tasks. In: *Lecture Notes in Computer Science*. Springer Verlag, pp 105–116, 10.1007/978-3-319-16220-1_8 (2015).

[50] Minervini M, Fischbach A, Scharr H, Tsaftaris SA (2016). Finely-grained annotated datasets for image-based plant phenotyping. Pattern Recognit Lett;.

